# Canolol Inhibits Gastric Tumors Initiation and Progression through COX-2/PGE2 Pathway in K19-C2mE Transgenic Mice

**DOI:** 10.1371/journal.pone.0120938

**Published:** 2015-03-17

**Authors:** Donghui Cao, Jing Jiang, Tetsuya Tsukamoto, Ruming Liu, Lin Ma, Zhifang Jia, Fei Kong, Masanobu Oshima, Xueyuan Cao

**Affiliations:** 1 Division of Clinical Epidemiology, First Hospital of Jilin University, Changchun, Jilin 130021, China; 2 Department of Diagnostic Pathology I, School of Medicine, Fujita Health University, Toyoake, Japan; 3 Department of Gastric and Colorectal Surgery, First Hospital of Jilin University, Changchun, Jilin 130021, China; 4 Cancer Research Institute, Kanazawa University, Kanazawa, Japan; The University of Texas MD Anderson Cancer Center, UNITED STATES

## Abstract

4-vinyl-2, 6-dimethoxyphenol (canolol) is an antioxidant phenolic compound extracted from crude canola oil. In current research, K19-C2mE transgenic mice, developing hyperplastic tumors spontaneously in the glandular stomach, were used to study the mechanisms involved in the anti-inflammation and anti-tumor effects of canolol. Tg mice receiving canolol diet had a reduced tumor incidence, to 41.2%, compared with Non-treatment Tg mice, 77.8% of which had gastric tumor (P=0.002). Besides that, the mean tumor diameter was decreased from 6.5mm to 4.5mm (P<0.001) after canolol administration. COX-2/PGE2 pathway is known to play pivotal role in inflammation-induced gastric tumorigenesis. The neutrophils and lymphocytes infiltration was suppressed significantly, and the mRNA levels of the proinflammatory cytokines COX-2, IL-1β and IL-12b were also downregulated in gastric mucosa. Additionally, immunohistochemical analysis showed that COX-2, EP2, Gαs and β-catenin, key factors involving in PGE2 signal transduction, were positive staining with higher H scores in Non-treatment Tg mice, while the expressions were suppressed significantly by 0.1% canolol (P<0.001). In addition, tumor-suppressor miR-7 was reactivated after canolol administration, and COX-2 was showed to be a functional target of miR-7 to suppress the tumor progression. In conclusion, canolol could inhibit the gastritis-related tumor initiation and progression, and the suppression effect was correlated with the blocking up of canonical COX-2/PGE2 signaling pathway and might be regulated by miR-7.

## Introduction

Gastric cancer remains the fourth most commonly diagnosed cancer and is the second leading cause of cancer related deaths worldwide. It has been shown that approximately 15–20% of malignant cancers are associated with chronic infection[1], and inflammatory responses play important roles in cancer development. Among various inflammatory networks involved in gastric carcinogenesis, the COX-2/PGE2 pathway was identified as a key player firstly[2]. The biological mechanisms of how this inflammatory pathway is involved in tumorigenesis is becoming a hot spot to study[3,4].

The cyclooxygenases (COX), COX-1 and COX-2, are key enzymes in the synthesis of prostanoids. COX-1 is considered to express constitutively and to maintain the integrity of the gastrointestinal mucosa, whereas COX-2 is an inducible enzyme and can generate PGs (prostaglandins) responsible for inflammatory responses[5]. The final conversion to PGE2 (prostaglandin E2) is mediated by microsomal prostaglandin E synthase mPGES-1. K19-C2mE transgenic mice, constructed by Oshima et al[6], could simultaneously overexpressed COX-2 and mPGES-1 in gastric mucosa epithelial cells, and developed hyperplastic tumor in the glandular stomach with heavy macrophage infiltrations. Then the transgenic mouse was recognized as the ideal model to investigate the precise role of COX-2/PGE2 pathway in gastric tumor initiation and progression.

Heterotrimeric guanine nucleotide-binding proteins (G proteins), consisting of α, β and γ subunits, function to relay information from cell surface G-protein-coupled receptors (GPCRs) to intracellular effectors in various transmembrane signaling pathways. The α subunit of G proteins are divided into four subfamilies: Gαs, Gαi, Gαq and Gα_12_. In mammals, most interest in G proteins was focused on the Gαs subfamily[7]. There are four GPCRs for PGE2, namely, EP1–4[8], and they are linked to different signaling pathway, whereas, EP2 can couple with Gαs to stimulate adenylyl cyclase (AC) and then increase intracellular cAMP level[9]. cAMP is thought to be the main intracellular second messenger of PGE2 signaling and can modulate the functional activity of macrophages and monocytes[10]. Besides the AC sideway, Gαs concomitantly binds Axin, and the binding leads to phosphorylation of glycogen synthetase kinase 3β (GSK3β) and caused the stabilization, nuclear translocation and transcriptional activation of β-catenin[11]. β-catenin is a key component of PGE2 signaling pathway, and the translocation and accumulation of β-catenin in nuclear combined with TCF/LEF transcription factor and influenced the downstream gene (i.e. COX-2, IL-8, VGFR) expression.

MicroRNAs (miRNAs) are a group of non-coding small RNAs (20–25 nt) known to negatively regulate gene expression by directly binding to the 3’-UTR of target mRNAs. Analyses of miRNA expression profiles indicate miRNAs are implicated in the pathogenesis of various diseases especially in cancer and correlated with tumor initiation, progression and metastasis[12], and miRNAs have been identified as potential candidates for novel diagnostic biomarkers or therapeutic targets of cancer. miR-7 has been proven to play a substantial role in hepatocellular carcinoma [13], breast cancer[14], and lung cancer[15]. In addition, miR-7 was found downregulated in gastric tumors in an inflammation-dependent manner in gastric cancer[16]. However, a functional link between miR-7 and the COX-2/PGE2 pathway has not been established.

Canolol, 4-vinyl-2, 6-dimethoxyphenol, a potent antioxidant phenolic compound extracted from crude canola oil. The analysis of canolol bioactivity showed its scavenging potency against ROO• is much higher than that of well-known antioxidants, such as α-tocopherol, vitamin C and β-carotene[17]. The oxidative responding molecules heme oxygenase-1 (HO-1) and 8-OHdG, relevant to carcinogenesis, were suppressed in mice receiving the canolol-containing diet[18,19]. Earlier study showed mutant formation of Salmonella and *E*. *coli* by ONOO- at physiological level was remarkably suppressed by canolol, which blocked strand break of bacteria by ONOO- in the presence of canolol[20]. Besides that, canolol could inhibit cell proliferation and induce cell apoptosis[21], and suppress the colitis-associated carcinogenesis[18]. The main object of our research was to examine the inhibitory effects of canolol on gastric tumor initiation and progression *in vivo*, and to further investigate the possible mechanisms involving PGE2 signal transduction via miRNA regulation.

## Material and Methods

### Animals

K19-C2mE transgenic (Tg) mice produced by Oshima et al.[6] were maintained by breeding male K19-C2mE Tg mice with female C57BL/6 N mice at the Animal Facility of Jilin University. C57BL/6 N is the most widely used inbred strain of laboratory mice due to the availability of congenic strains, easy breeding, and robustness. For genotyping of each mouse, DNA samples were extracted from the tails of 4 weeks old Tg mice using Multisource Genomic Miniprep DNA (Axygen) and subjected to PCR as reported in[22].

### Chemicals

Canolol (molecular weight, 180 Da), with >95% purity, was kindly provided by Dr. Masae Tatematsu from Aichi Cancer Center Research Institute and Prof. Hiroshi Maeda from Sojo University. In our study, it was mixed with 2, 6-di-tert-butyl-4-methylphenol [butylated hydroxytoluene (BHT), Sigma] at final concentration of 300pm. BHT at this concentration was added to protect canolol from oxidation[6], and had no significant therapeutic effect on cancer[19]. The preparation in solid form or solution was sealed under helium or nitrogen, and stock solution in ethanol was kept at -80°C.

### Diets

The modified AIN93G diet containing 0.1% canolol and 0.3ppm BHT was used in this study as previously described[19]. Canolol was first dissolved in soybean oil and then mixed into the diet to the concentration of 0.1%. The control diet contained the same components but no canolol. The diets were sealed and stored at -30°C; they were given daily after being thawed. Each day, leftovers from the previous day’s feeding were measured, and new food was provided to replace the eaten amount.

### Experimental design

**Fig 1:**
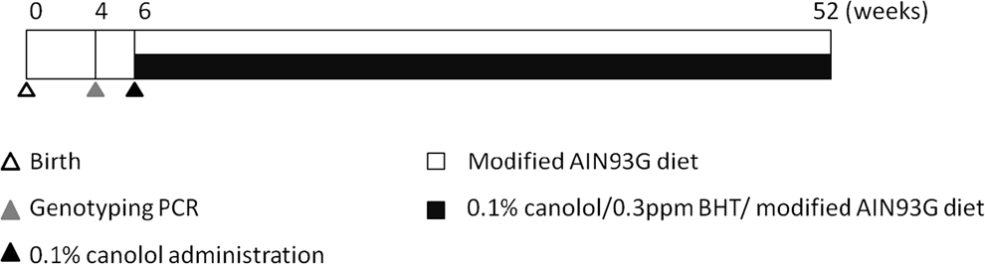
Experiment design in this study. 4 weeks old mice were subjected to genotyping PCR, after identified, 6 weeks old Tg mice were randomly divided into Non-treatment group and Canolol-treated group. All the mice were fed with modified AIN93G diet with(out) 0.1% canolol till 52 weeks.

The experimental design in this research was shown in Fig. 1. After genotyping when Tg mice were 4 weeks old, the Tg mice were randomly divided into Non-treatment group and Canolol-treated group. In Canolol-treated group, 0.1% canolol contained in the modified AIN93G diet was oral administrated till 52 weeks, while the Tg mice in Non-treatment group were used as control.

All mice were housed in plastic cages with hardwood chips in an air-conditioned room with 12 h light–12 h dark cycle and were allowed free access to food and water. The water containing 0.1% canolol was changed on the next day to prevent excessive oxidation and the leftover were measured. At the end of the experiment, all surviving mice were sacrificed under deep anesthesia using diethyl ether and all efforts were made to minimize suffering. The study was approved by the Animal Care Committee of Jilin University.

### Tissue collection

The stomach was resected and cut along the greater curvature. Pair-matched tumor and Non-tumor were separated from Tg mice, and the size of a gastric tumor was measured using vernier caliper as the maximum diameter. Shortly after surgical resection, all the samples were divided into two parts, one part was immediately snapfrozen in liquid nitrogen and stored at -70°C for RNA extraction. Another part was fixed in 10% neutral buffered formalin, and wax embedded for HE and immunohistochemical analysis.

### HE staining and Immunohistochemistry assessment

Tissue sections were stained with hematoxylin—eosin for histological analysis. The glandular mucosa was examined histologically for any inflammatory and epithelial changes. Active chronic gastritis was characterized by infiltration of neutrophils and lymphocytes. The degree of inflammatory changes was graded on a 4-point scale (0 to 3; 0, normal; 1, mild; 2, moderate; 3, marked gastritis) according to the criteria modified from the updated Sydney System.

Serial sections were also stained immunohistochemically with antibodies against EP2 (Cayman Chemical, Ann Arbor, MI, USA), COX-2, β-catenin, and Gαs (Santa Cruze, CA, USA). The expressions of above antibodies were categorized by doing an H score, which combines intensity of staining in each cell and percentage of stained cells[23]. In brief, 4 μm tissue section was assigned a staining intensity score from 0 to 3 (I0 [normal], I1 [mild], I2 [moderate], and I3 [marked]), and a percent of stained tumor cells that was recorded in 5% increments from a range of 0 to 100 (P0, P1–3). A final H score (range 0–300) was obtained by adding the sum of individual scores obtained for each tissue (H score = I1×P1 +I2×P2+ I3×P3).

### Protein extraction and Western blot analysis

The proteins located in cytoplasm and nucleus of tumor tissues were extracted independently using the Nuclear and Cytoplasmic Extraction Kit (Kangwei, China). Proteins were separated by 15% SDS-PAGE and transferred onto PVDF membranes. After being blocked in 5% nonfat milk, the proteins were probed with human-origin β-catenin polyclonal antibodies[24] (1:1,000, Santa Cruze, sc-1496), mouse-origin β-actin monoclonal antibody[25] (1:1,000, Santa Cruze, sc-47778) and then incubated with HRP labeling goat anti—human and anti-mouse IgG (1:1,000, Beyotime), and then detected with ECL reagents (Thermo Fisher Scientific) using Molecular Imager ChemiDox XRS+ Imaging System.

### RNA isolation and qRT-PCR quantification

Total RNA rich of miRNA of tumor and Non-tumor tissues were extracted using the mirVana miRNA Isolation Kit (Ambion, TX, USA) following the manufacturer’s guidelines. Once extracted, RNA concentrations and quality were determined with a NanoDrop ND-1000 spectrophotometer (NanoDrop Technologies, DE, USA) and agarose gel analysis. Thereafter, RNA was reversed-transcribed using TaqMan MicroRNA Reverse Transcription Kit (ABI) for miR-7 detection and cDNA synthesis kit (Roche) for COX-2, mPGES-1, Gαs, IL-1β, IL-6 IL-12b and HO-1. The primers sequences were listed at S1 Table. A tube with no reverse transcriptase was included to control for any DNA contamination.

The expression of miR-7 and the other genes was assayed on ABI7500 real-time PCR detection system and normalized with snoRNA202 and GAPDH respectively. The 2^-ΔΔCt^ method for relative quantization was used to determine miRNA expression. As the analysis of miR-7 expression, the Ct is the fractional cycle number at which the fluorescence of each sample passes the fixed threshold. The ΔCt was calculated by subtracting the Ct of snoRNA202 from the Ct of the miR-7. The ΔΔCt was calculated by subtracting the ΔCt of the reference sample (paired nontumorous tissue) from the ΔCt of each tumorous sample. Fold change was determined as 2^-ΔΔCt^.

### Cell culture and vector construction

HEK293T was obtained directly by Shanghai GeneChem (Lot number: GCPC53508) and cultured in DMEM medium (Hyclone) containing 10% heat-inactivated fetal bovine serum (Hyclone) in an incubator (5%CO_2_) at 37°C (Thermo). According to the prediction of miR-7 target using TargetScan (http://www.targetscan.org/) and miRanda-mirSVR (http://www.microrna.org/), two regions, position 86–92 and 1111–1118 of COX-2 were combined with miR-7 seed region possibly. Then both regions of COX-2 3’-UTR were cloned and constructed independently into pGL3 vector. In addition, the miR-7 up eukaryotic expression plasmid and the mutants of COX-2 3’-UTR plasmids were also supplied by Shanghai GeneChem.

### Cell transfeciton and Dual—luciferase reportor assay

HEK239T cells in logarithmic growth phase were seeded in 24-well plate, and were cotransfected with 1μg miR-7 and COX-2 plasmid using lipo 2000 (Invitrogen) as the manual protocol. After 24 hours of transfection, the cells were harvested for luciferase activity assay using the dual-luciferase reporter assay kit (Promega). The renilla luciferase activities were used to normalize the both transfection and harvest efficiencies. The firefly luciferase activity of each sample was normalized to the Renilla luciferase activity.

### Statistics

All analyses were performed using SPSS version 10.0 (SPSS Inc, USA). Data were evaluated using one-way ANOVA. A value of P < 0.05 was considered significant and P<0.01 was very significant.

## Results

### Suppressive effects of canolol on tumor progression in Tg mice

70 male mice were identified as Tg mice using genotyping PCR, and then 34 Tg mice were chosen to be administrated with 0.1% canolol, while the other 36 Tg mice were fed with basal diet and used as control. To examine the effects of canolol on the tumor initiation, the tumor incidence was calculated in both groups. As showed in Table 1, Tg mice receiving canolol diet had a reduced tumor incidence, to 41.18% (14/34), compared with Non-treatment Tg mice, 77.78% (28/36) of which had gastric tumor (P = 0.002).

**Table 1:** The tumor incidence in Non-treatment group and Canolol-treated group.

Group	mice bearing tumors		mice without tumors		P value
	Number	Incidence	Number	Incidence	
**Non-treatment**	28	77.78%	8	22.22%	0.002
**Canolol-treated**	14	41.18%	20	58.82%	

**Fig 2:**
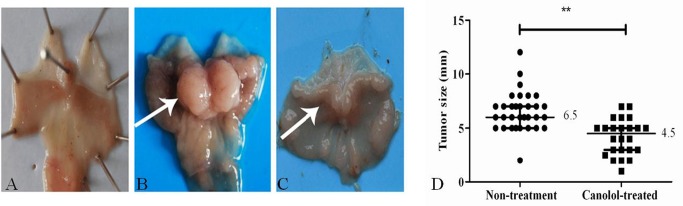
The macroscopic photograph in wild mice (A), Non-treatment Tg mice (B), Canolol-treated Tg mice (C), and the scatter plot of the tumor size (D). The arrows indicate gastric tumors occurred in Tg mice. Bold horizontal bar indicates the median with interquartile range. **, P<0.01.

After surgical resection, we found significant differences between the tumors existed in Non-treatment Tg group and Canolol-treated Tg group, and the macroscopy photos were showed in Figs. 2A–2C. Besides that, the tumors size in both groups was measured using vernier caliper, the mean diameter of tumor in Tg group was 6.5mm, while it decreased into 4.5mm after 0.1% canolol administration (P<0.001) (Fig. 2D).

### Canolol exhibits anti-inflammatory activity in Tg mice

**Fig 3:**
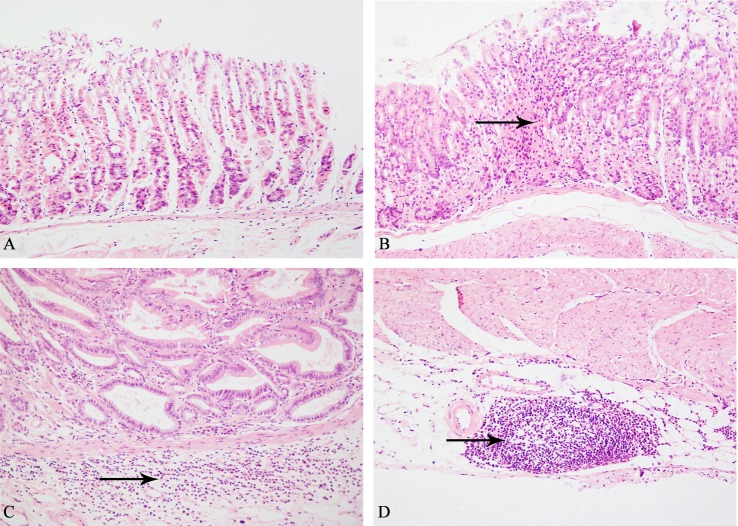
Histological examination showed the grades of gastritis. The normal gastric epithelial in wild type mice (grade 0, A), moderate (grade 2, C) and marked (grade 3, D) gastritis in Non-treatment Tg mice, and the alleviation of inflammatory responses in Canolol-treated Tg mice (grade 1, B). The arrow indicates mononuclear cell infiltration into the gastric glands or lymphoid follicle formed in submucosa. HE×20

It is considered that chronic active gastritis is an important risk factor of gastric carcinogenesis[26]. To investigate the preventive effects of canolol on inflammatory responses occurred in Tg mice, mucosal inflammation was analyzed on HE stained sections, and grade 0–3 were classified according to histopathological criteria. All gastric mucosal specimens from wild type mice had no malignant lesions (grade 0, Fig. 3A), and numerous neutrophils and lymphoplasmocytic cells were found infiltrating in the gastric glands and submucosa (grade 2, Fig. 3C), and lymphoid follicle was formed apparently in Non-treatment Tg mice (grade 3, Fig. 3D), however, Tg mice received 0.1% canolol administration showed suppressed mononuclear cell infiltration in the mucosa (grade 1, Fig. 3B).

### Canolol inhibited the expression of COX-2/PGE2 pathway proteins in Tg mice

**Fig 4:**
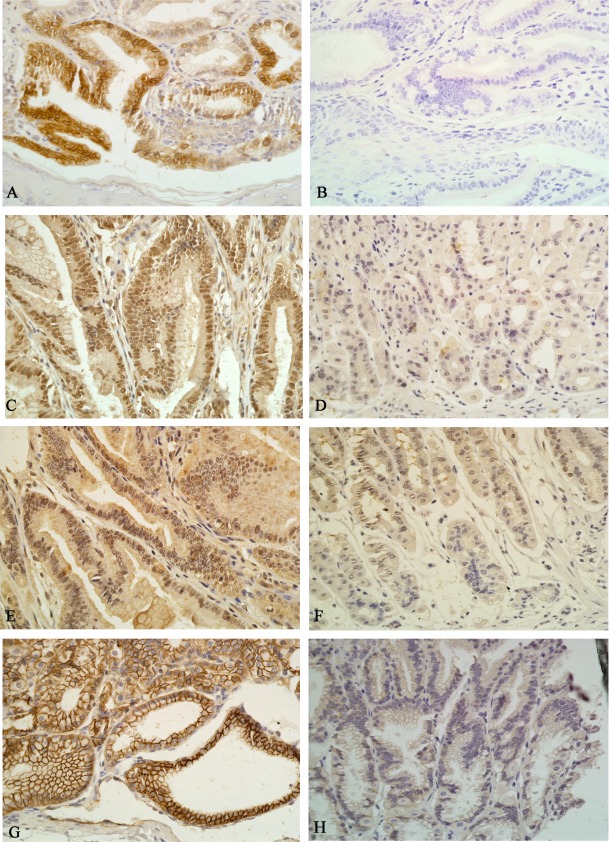
Immunohistochemical staining of COX-2 (A, B), EP2 (C, D), Gαs (E, F) and β-catenin (G, H) in Non-treatment mice (left panel) and the Canolol-treated mice (right panel). Note that the intensity of COX-2, EP2, Gαs and β-catenin immunoreactivity in the Canolol-treated Tg gastric mucosa is weaker than that in Non-treatment Tg group. IHC×40.

**Fig 5:**
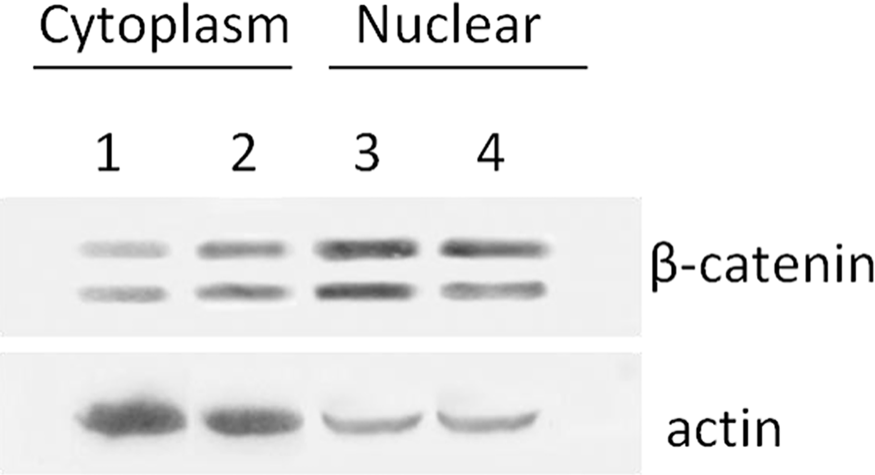
The β-catenin expression in cytoplasm and nuclear in Non-treatment (1&3) and Canolol-treated (2&4) gastric tumor tissue. Note the β-catenin nuclear accumulation was decreased in Canolol-treated groups (Lane 4) compare to Non-treatment groups (Lane 3).

To investigate whether COX-2/PGE2 signaling pathway was the target of canolol, the expression levels of COX-2, EP2, Gαs and β-catenin, four kinds of pivotal factors in the pathway were analyzed using immunohistochemical stainning in tumors of Non-treatment Tg mice and Canolol-treated Tg mice, in addition, H score was generated according the stain intensity of each cell and the percentage of stained cell. The IHC results showed that COX-2 (Fig. 4A), EP2 (Fig. 4C), Gαs (Fig. 4E) and β-catenin (Fig. 4G) showed ‘nuclear/cytoplasmic’ and ‘membranous’ positive expression with higher H scores in almost all tumors in Non-treatment Tg mice, while after 0.1% canolol administration, COX-2 mainly showed cytoplasmic and membranous expression and no nuclear expression (Fig. 4B), EP2 mainly located on membrane (Fig. 4D), Gαs mainly expressed in nuclear or cytoplasm (Fig. 4F), and β-catenin nuclear accumulation was significantly alleviated (Fig. 4H). Besides that, the H scores demonstrated 0.1% canolol could suppress COX-2, EP2, Gαs and β-catenin expression significantly (P<0.01, Table 2). In addition, Western blot analysis also showed that the β-catenin level was elevated in nucleus, and the nuclear accumulation was downregulated in canolol-treated groups compare to Non-treatment groups. Meanwhile, there were not significantly differences in β-catenin expression levels of cytoplasm between two groups (Fig. 5).

**Table 2:** H score calculated on the basis of immunohistochemical staining of COX-2, EP2, Gαs and β-catenin.

Proteins	K19-C2mE (Tg) N = 33	Canolol-treated N = 14	P value
COX-2	165(150–180)	145(125–160)	0.002
EP2	170(148–190)	100(75–108)	<0.001
Gαs	225(200–260)	150(133–195)	<0.001
β-catenin	190(168–200)	150(98–173)	0.001

H score was presented as the median with quantile range.

### Expression of COX-2/PGE2 signal pathway genes and miR-7 in gastric tumor

**Fig 6:**
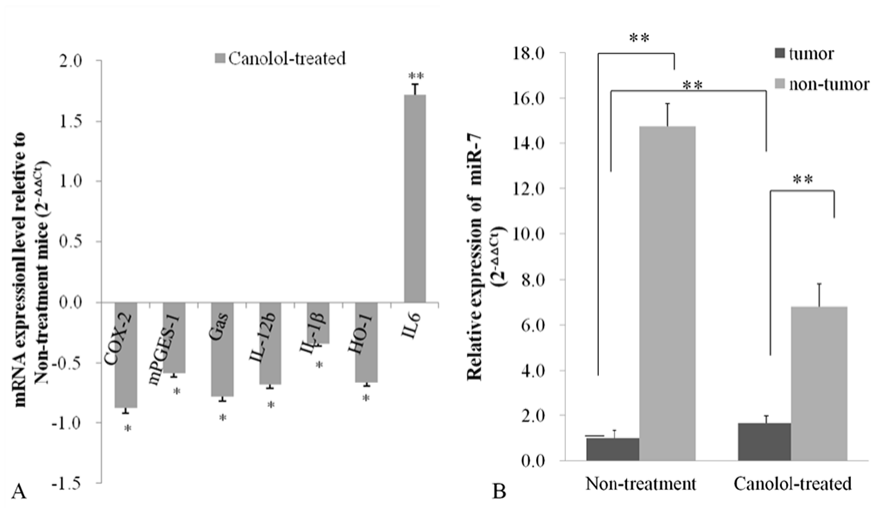
The effects of canolol on the COX-2/PGE2 pathway genes (A) and miR-7 expression (B). The studied genes involved proinflammatory cytokines (IL-12b, IL-1β, IL-6), oxidative responding gene HO-1, PGE2 synthases genes (COX-2, mPGES-1) and Gαs that relays PGE2 signal into cytoplasm. The horizontal bar in B indicates the 1.0 *, P<0.05; * *, P<0.01.

**T**o deeply investigate the chemopreventive mechanisms of canolol, the mRNA expression levels of representative oxidative inflammatory molecules, i.e. COX-2, mPGES-1, Gαs, IL-1β, IL-6, IL-12b and HO-1 were analyzed using qRT-PCR. The results showed that COX-2, mPGES-1, Gαs, IL-1β, IL-12b were downregulated significantly (Fig. 6A). As to COX-2 and Gαs, two key factors in COX-2/PGE2 signaling pathway, the mRNA levels were decreased to 87.47% and 78.33% respectively, which were consistent with the decreased protein levels in IHC results (Figs. 4A–4D). mPGES-1, highly expressed in Non-treatment Tg mice, were also decreased to 58.51% after canolol administration. In addition, the expression level of IL-1β and IL-12b, two kinds of inflammatory cytokines, were decreased to 33.96% and 68.34% respectively. Besides that, HO-1, a major antioxidative antiapoptotic molecule reflecting oxidative cellular stresses, was also found decreased to 66.47% after canolol administration.

To further study whether miRNAs participate in the COX-2/PGE2 signal transduction, the relative expression level of miR-7 was analyzed using qRT-PCR. The results showed that miR-7 expression level was significantly higher in Non-tumor normal mucosa (P<0.01), which suggested miR-7 might play tumor suppressor role and its activity was inhibited in gastric tumors. However, in the Tg mice receiving canolol administration, the miR-7 expression was reactivated significantly (P<0.01) (Fig. 6B).

### COX-2 was a functional target of miR-7

**Fig 7:**
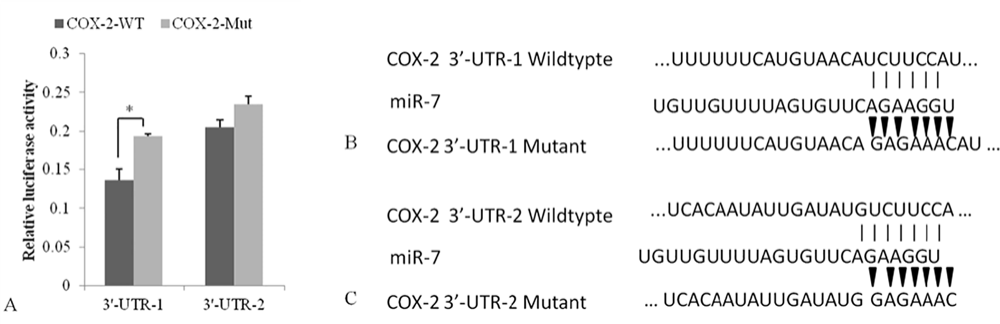
COX-2 is the direct target of miR-7. The relative luciferase activity was reduced significantly in 3’-UTR-1 but not in 3’-UTR-2 (A). miR-7 and its putative two binding sequence in the 3’-UTR of COX-2, 86–92 sites (B) and 1111–1118 sites (C). The mutants of 3’-UTR were indicated by the arrows. *, P<0.05

To elucidate the underlying mechanisms of the suppressive effects of miR-7 on tumor progression, TargetScan (http://www.targetscan.org/) and miRanda-mirSVR (http://www.microrna.org/) were used to predict the miR-7 targets, and COX-2, a pivotal factor in the COX-2/PGE2 signal pathway, was found to be the possible target. In addition, two regions, position 86–92 (3’-UTR-1) (Fig. 7B) and 1111–1118 (3’-UTR-2) (Fig. 7C) of COX-2 have putative miR-7-binding elements. We found that miR-7 inhibited the luciferase activity of a reporter containing the wild-type COX-2 3’-UTR-1 but not that of 3’-UTR-2 after the dual-luciferase reporter assay (Fig. 7A). The results showed that COX-2 was a functional target through the combination with 3’-UTR-1.

## Discussion

K19-C2mE transgenic mice model, which simultaneously expressed COX-2 and mPGES-1 in gastric epithelial cells and could develop hyperplastic tumors spontaneously in the glandular stomach, was used in this study[22]. In our previous research, canolol as the potential COX-2 inhibitor could inhibit tumor cell growth while induce cell apoptosis[21], but the mechanisms should be deeply studied.

The wildly used drug in gastric tumor treatment is nonsteroidal anti-inflammatory drug (NASID), and the best-known inhibition pathway of NASID is the biosynthesis of PGE2. PGE2 plays pivotal roles in angiogenesis[27]; cell survival[28] and activation of the EGFR [29]. COX-2 and mPGES-1, key enzymes of PGE_2_ synthases, were overexpressed in Non-treatment Tg mice, while after canolol administration, the mRNA expression of COX-2 and mPGES-1 were suppressed significantly (Fig. 6A), and the IHC analysis of COX-2 showed that the suppression effect also exist in protein translation procedure (Table 2, Figs 4A and 4B). The results suggested canolol as a new COX-2 inhibitor had the potential to be developed as a new anti-tumor drug.

Besides COX-2 and mPGES-1, EP2, G protein, AC, PKA, Axin, cAMP, β-catenin were also key regulators participating in PGE2 transduction[7]. When PGE2 was generated, Gαs subfamily of G protein can relay the stimulus information into cytoplasm, and EP2, one of the G-protein coupled receptors (GPCRs), can generate one or more intracellular messengers i.e. AC and Axin. Previous studies found EP2/Gαs coupled with PGE2 exerted proneoplastic effects by stimulating critical oncogenic pathways, such as the GSK3β/β-catenin pathway[30] and cAMP/PKA pathway[31] in colon[8], lung[32] and prostate cancer[33]. The IHC results in our study showed that EP2, Gαs and β-catenin were strong stained in membrane, cytoplasm or nucleus in Non-treatment Tg mice, while after canolol administration, the intensity of EP2 (Figs 4C and 4D) and Gαs (Figs 4E and 4F) was decreased, and the nucleus accumulation of β-catenin was alleviated significantly (Figs 4G, 4H and 4). And these results suggested a prominent pathway that canolol could inhibit PGE2 signaling transduction to cytoplasm through Gαs-coupled receptor EP2, and then blocked the nucleus translocation of β-catenin.

It was widely accepted inflammation had an important role in cancer development. Previous studies have showed that EP2 could promote cell growth and invasion through upregulation of the tumor-promoting inflammatory cytokines, such as IL-1β and IL-6[34]. And promoter analysis of EP2 found several consensus sequences that relevant to inflammatory stimuli[35]. Consistent with the conclusion, the expression levels of EP2, COX-2, mPGES-1, IL-1β, IL-12b and HO-1 were downregulated simultaneously by canolol in our research (Figs. 4A, 4D and 6). Besides that, HE staining analysis showed the neutrophils and lymphoplasmocytic cells were found infiltrated heavily into gastric glands and submucosa and formed lymphoid follicle in Non-treatment Tg mice (Figs. 3C and 3D), and the gastritis degree was graded as 2–3. However, the degree of gastritis decreased to grade 1 in canolol-treated Tg mice (Fig. 3B). Our previous study on Mongolian gerbils also showed 0.1% canolol administration could effectively suppressed *H*. *pylori*-associated chronic gastritis and gastric tumor growth[19]. It indicated that canolol could block the nucleus translocation of β-catenin, and suppress the gene transcription of proinflammatory cytokines through the combination with transcription factors CREB and TCF, and then inhibit inflammatory-related gastric tumorigenesis.

Accumulating evidences showed miRNAs played essential roles in tumor initiation, progression and metastasis, and miR-7, one of the inflammation-related miRNAs in gastric tumor, was delineated to be a potential tumor suppressor[16]. To further explore the mechanism of anti-inflammation and anti-tumor effects of canolol, the miR-7 expression was detected using qRT-PCR. Briefly, miR-7 was silenced expressed in tumor tissue in Non-treatment Tg mice, while the miR-7 expression was reactivated after canolol administration (Fig. 6B). The activation of miR-7 and the suppression of COX-2 in the Canolol-treated Tg group were observed simultaneously in our research, and COX-2 was showed a functional target of miR-7 (Fig. 7). The results suggested that miR-7 downregulated the gene transcription of COX-2 through the incomplete combination with 3’-UTR region, and interrupted the PGE2 synthesis, and then blocked the whole signaling pathway. It suggested that miRNAs regulation, an important epigenetic modification, might play some key roles in suppression effect of canolol on gastric tumor initiation and progression.

In conclusion, canolol, an anti-oxidant natural product without side effects, could inhibit gastric tumor initiation and progression through blocking the COX-2/PGE2/EP2 signaling transduction pathway. Briefly, canolol could suppress the nucleus translocation of β-catenin through Gαs coupled receptor EP2, and lead to the inhibition of gene transcription of oncogenic genes, and the inhibitory effect might correlate with miRNA regulation. To clearly elucidate the entire network of inhibitory mechanisms of canolol and how miRNA correlate with the canonical pathway in gastric tumorigenesis, mRNA and miRNA expression arrays should be performed in Non-treatment Tg mice and Canolol-treated Tg mice.

## Supporting Information

S1 Table The sequences of primers used in qRT-PCR. (DOCX)
