# Supplementary material for: Canolol Inhibits Gastric Tumors Initiation and Progression through COX-2/PGE2 Pathway in K19-C2mE Transgenic Mice
Source: PLoS One. 2015 Mar 17;10(3):e0120938. doi: 10.1371/journal.pone.0120938 (PMC4363315; doi:10.1371/journal.pone.0120938)
Supplement: S1 Table — (DOCX) [file pone.0120938.s001.docx]

| Primer |  | Sequences | Length (bp) | Product (bp) |
| --- | --- | --- | --- | --- |
| GAPDH | F | TGTGTCCGTCGTGGATCTGA | 20 | 150 |
|  | R | TTGCTGTTGAAGTCGCAGGAG | 21 |  |
| COX-2 | F | TGAGCAACTATTCCAAACCAGC | 22 | 74 |
|  | R | GCACGTAGTCTTCGATCACTATC | 23 |  |
| mPGES-1 | F | GGATGCGCTGAAACGTGGA | 19 | 133 |
|  | R | CAGGAATGAGTACACGAAGCC | 21 |  |
| Gαs | F | CGCGAGGCCAACAAAAAGAT | 20 | 170 |
|  | R | GTGGCCTTCTCACTATCTCCG | 21 |  |
| Il-1β | F | GCAACTGTTCCTGAACTCAACT | 22 | 89 |
|  | R | ATCTTTTGGGGTCCGTCAACT | 21 |  |
| Il6 | F | CCTCTCTGCAAGAGACTTCCAT | 22 | 126 |
|  | R | ACAGGTCTGTTGGGAGTGGT | 20 |  |
| Il-12b | F | TGGTTTGCCATCGTTTTGCTG | 21 | 123 |
|  | R | ACAGGTGAGGTTCACTGTTTCT | 22 |  |
| HO-1 | F | AAGCCGAGAATGCTGAGTTCA | 21 | 100 |
|  | R | GCCGTGTAGATATGGTACAAGGA | 23 |  |

F：Forward; R：Reverse
